# 

**Published:** 1861-08

**Authors:** 


☞ The American Dental Convention will hold its seventh annual session at New Haven, Conn., commencing on Tuesday, the sixth day of August next, at 10 A. M.

It is desirable at this period in dental history to place the profession on a firm public basis. While the highest type of integrity shall characterize our professional efforts in our home labors, let us faithfully endeavor, even at personal sacrifices, to give character to the Annual Dental Convention, by contributing in person to the fund of knowledge obtained by hours of patient study and weary toil. Let the Convention be made worthy the appellation of "Sisterhood" to the American Medical Society. Although our field is not so varied as her's, there is ample scope for obtaining an advanced position, which will give increased efficiency, permanency and character to the dental profession. With "Excelsior" for our motto, let us earnestly coöperate in making our's second to none of the learned professions in the land.

I. J. Wetherbee, *Cor. Sec'y.*

### DENTAL REGISTER, OF THE WEST.

Issued Monthly, at $3 a year in advance.

DEVOTED TO THE INTERESTS OF THE DENTAL PROFESSION.

**Edited by J. TAFT and GEO. WATT.**

The Volume commences in January and closes in December.

The Register being issued monthly is always fresh, therefore indispensable to the practitioner who desires to keep posted up with the new inventions and improvements which are daily developed. Neither expense nor effort will be spared to give value and variety to its contents. Articles will be illustrated with well executed engravings, whenever they will add to the interest or assist in explanation.

Its extensive circulation and frequency of issue makes it the BEST DENTAL ADVERTISING MEDIUM in the United States.

☞ CONTRIBUTIONS SOLICITED.

Address Original Communications to **Geo. Watt, Xenia, O.**

Exchanges to **J. Taft,** or **Dental Register, Cincinnati, O.**

Business Communications, to

**JNO. T. TOLAND, Publisher and Proprietor,**
38 West Fourth Street, Cincinnati, O.

Communications must reach the Editor as early as the 15th, to insure insertion in the next issue.

